# Impact of Climate Change on Voltinism and Prospective Diapause Induction of a Global Pest Insect – *Cydia pomonella* (L.)

**DOI:** 10.1371/journal.pone.0035723

**Published:** 2012-04-23

**Authors:** Sibylle Stoeckli, Martin Hirschi, Christoph Spirig, Pierluigi Calanca, Mathias W. Rotach, Jörg Samietz

**Affiliations:** 1 Agroscope Changins-Wädenswil Research Station ACW, Wädenswil, Switzerland; 2 Federal Office for Meteorology and Climatology MeteoSwiss, Zürich, Switzerland; 3 Agroscope Reckenholz-Tänikon Research Station ART, Zürich, Switzerland; 4 University of Innsbruck, Institute for Meteorology and Geophysics, Innsbruck, Austria; Pacific Climate Impacts Consortium, Canada

## Abstract

Global warming will lead to earlier beginnings and prolongation of growing seasons in temperate regions and will have pronounced effects on phenology and life-history adaptation in many species. These changes were not easy to simulate for actual phenologies because of the rudimentary temporal (season) and spatial (regional) resolution of climate model projections. We investigate the effect of climate change on the regional incidence of a pest insect with nearly worldwide distribution and very high potential for adaptation to season length and temperature – the Codling Moth, *Cydia pomonella*. Seasonal and regional climate change signals were downscaled to the hourly temporal scale of a pest phenology model and the spatial scale of pest habitats using a stochastic weather generator operating at daily scale in combination with a re-sampling approach for simulation of hourly weather data. Under future conditions of increased temperatures (2045–2074), the present risk of below 20% for a pronounced second generation (peak larval emergence) in Switzerland will increase to 70–100%. The risk of an additional third generation will increase from presently 0–2% to 100%. We identified a significant two-week shift to earlier dates in phenological stages, such as overwintering adult flight. The relative extent (magnitude) of first generation pupae and all later stages will significantly increase. The presence of first generation pupae and later stages will be prolonged. A significant decrease in the length of overlap of first and second generation larval emergence was identified. Such shifts in phenology may induce changes in life-history traits regulating the life cycle. An accordingly life-history adaptation in photoperiodic diapause induction to shorter day-length is expected and would thereby even more increase the risk of an additional generation. With respect to Codling Moth management, the shifts in phenology and voltinism projected here will require adaptations of plant protection strategies to maintain their sustainability.

## Introduction

Temperate regions are and will be affected by global warming, with more or less pronounced changes depending on greenhouse gas emission scenario [1]. In any case projected warming will affect plant and animal species and communities with respect to physiology (e.g. tree growth), species distributions (e.g. range-shifts), phenology (e.g. flowering season or voltinism), and evolutionary adaptations (e.g. life-history) [2], [3], [4], [5], [6]. Terrestrial arthropods are suspected to respond strongly and quickly as their development, reproduction and distribution are strongly determined by temperature (ectothermy) [7]. In many insect species both an earlier beginning and prolongation of the season associated with the recent warming have already been documented [8]. Future shifts in species distribution poleward in latitude and upward in elevation are therefore expected as a consequence of climate change [9]. Such shifts in phenology and distribution ranges may also induce changes in life-history traits. An example is the shift of photoperiodic diapause induction to shorter day-lengths in the pitcher plant mosquito (*Wyeomyia smithii* Coquillett) [10].

The Codling Moth, *Cydia pomonella* (L.) (Lepidoptera: Tortricidae), is considered as the key pest in apple orchards worldwide except for parts of Eastern Asia and Western Australia [11]. The level of fruit infestation can reach 100% in untreated apple orchards [12]. Injury of at most 1% damaged fruit can be tolerated due to the direct fruit attack. The Codling Moth has a facultative diapause with increasing number of generations towards the southern latitudes [13]. In general the species is univoltine north of the isotherm +20°C for July [13]. The number of generations depends on season length and climate, i.e. on the possibility to complete a certain generation successfully and thereby to profit from the last generation in terms of evolutionary fitness. Photoperiod is the signal for regulation of the life cycle in multivoltine Codling Moth populations with a facultative diapause. Codling Moth populations are adapted to the local light regime by their specific critical photoperiod of diapause induction. All larval stages are photosensitive (most susceptible in 3^rd^ and 4^th^ stage [13]) and 5^th^-instar larvae enter diapause when photoperiod in later summer shortens below the critical photoperiod which is, for example, 16 h light at 47° northern latitudes [13]. The critical photoperiod increases with increasing latitude and thereby shortens the development phase in time according to the lower seasonal temperatures.

Due to its flexibility in life-history adaptation to season length and temperature, Codling Moth is an ideal example to study the response of insect phenology to global warming. The formation of additional generations has already been assumed in early assessment of climate change impact on biological systems [14]. This is even more important because already under present climate conditions a multi-level plant protection strategy is necessary for the control of the Codling Moth [12], [15] and changing phenology implies the necessity to adapt control methods as a basis of sustainable plant protection.

Hitherto the analysis of detailed insect phenologies with climate change scenarios at the local scale was limited by the low spatial and temporal resolution of the climate forecasts. However, recent attempts have shown that downscaling regional climate simulations to the temporal and spatial scale of pest habitats and life cycles can be used to overcome those drawbacks [16]. Accordingly in our study the assessment was carried out in two steps. First we used a stochastic weather generator (WG) in combination with a re-sampling approach to provide hourly synthetic weather data for ten meteorological stations in Switzerland. In a second step we used the Codling Moth model, as implemented in the SOPRA forecasting system [17] to create statistically valid Codling Moth phenologies for present and future climate. The resulting data sets were used to evaluate the impact of climate change on the Codling Moth life cycle across Switzerland and to determine voltinism and prospective photoperiodic diapause induction as critical life-history components to forecast future pest phenologies.

## Materials and Methods

### Simulation of Codling Moth development

The effect of climate change on Codling Moth was evaluated using the pest phenology model implemented in the forecasting system SOPRA for the sustainable management of orchard pests [17]. All SOPRA models were based on time-varying distributed delay routines on hourly basis [18], [19]. They simulated the proportion of life stages in the population with a temporal resolution of one day in the output. Due to the distributed delay approach, life stages were fully overlapping without limitations to the number of stages. Laboratory experiments using different temperature regimes were established to approximate the underlying linear and nonlinear relationships between temperature and individual development times [17]. Nonlinear functions were used for reproductive rates and survival of adults. The SOPRA Codling Moth model was driven by solar radiation, air and stem temperature on hourly basis. Stem temperature was approximated for hibernating larvae and pupae. Stem temperature was simulated from air temperature and solar radiation on the basis of the seasonal azimuth angle of the sun and light extinction of the vegetation [17]. To account for major changes in voltinism, the original SOPRA time-varying distributed delay routine was extended, so that it simulates the frequency distribution of an additional third following generation. The switch between univoltine and polyvoltine life cycle of Codling Moth was determined by a day-length signal in nature and accordingly by a latitudinally adapted day of year (DOY) in the model, which switches further development to diapause. For model validation, simulated emergence from hibernation sites and adult flight field observations (adult male moth trap catches) were considered [17] (Figs. S1 and S2).

For the assessment, voltinism was first analysed without changes in photoperiodic diapause induction. Photoperiodic diapause induction at 47° northern latitude was fixed at 16 h day-length, which corresponds to DOY 211 (end of July). Shorter day-lengths serve as a signal to larvae to diapause and to pupate next spring. Second, we simulated an evolutionary shift in photoperiodic diapause induction to a shorter day-length, i.e. a local adaptation to the extension of season length, which is to be expected with global warming. To do so, diapause induction was delayed stepwise (five-day) until beginning of September (DOY 246). This five-week delay in photoperiodic diapause induction corresponds to a decrease in day-length signal from 16 h to 13 h. DOY 246 was selected as diapause induction endpoint as relative phenology was similar compared without any diapause induction. Present climate conditions in southern California selected for Codling Moth populations that enter diapause when the day-length is shorter than 13 h [20].

The SOPRA output provides relative phenology for all relevant stages of the life cycle and is initialized with the pupae of the overwintering generation (100%). Then, the adult flight and oviposition of the overwintering generation is simulated. Egg development, larvae, pupae adult flight and oviposition are separated for the subsequent first, second and third following generations. For analysis, important stages such as adult flight, oviposition and larval emergence start, as well as peak larval emergence were considered. Codling Moth phenology of present and future climate was compared by means of changes in the occurrence of important stages (i.e., as changes in DOY), potential risk of a second and third generation, extent of relative phenology (magnitude), length of important stages, and overlapping generations. The risk for the development of a second generation was based on the probability of a first generation adult flight start (1% relative phenology) and of a second generation larval emergence start (2% relative phenology). Similarly the risk for the development of a third generation was based on the probability of a second generation adult flight start and of a third generation larval emergence start. The probability is based on 100 years of synthetic hourly weather series (see climate change scenarios). The magnitude of the larval stage was assessed by extent of relative phenology (peak as well as the 45% relative larval emergence). The length of oviposition and larval emergence (first and second generation), as well as first generation pupal development and adult flight, was measured in DOY between first occurrence and peak relative phenology. Peak relative phenology was selected as endpoint to measure the length of the population build-up and exclude confounding effect as e.g. diapausing larvae. The overlap of overwintering and first generation adult flight, as well as the overlap of first and second generation larval emergence was assessed in DOY. The number of overlapping DOY was based on presence/absence of the population within a specific event during the whole season.

### Development of climate change scenarios

Climate change scenarios provide basic information about changes of relevant climate parameters for the simulation of insect phenology [1]. For Europe, the highest spatial resolution available from regional climate model simulations is provided by the multi-model runs carried out in the context of the EU-FP6 project ENSEMBLES [21]. At 25 km horizontal resolution, these simulations are based on the A1B SRES greenhouse gas emission scenario [1] and were already used to obtain seasonal and regional probabilistic climate change signals for Switzerland [22]. Here, we restricted our attention to the median signals of the multi-model ensemble (Table 1).

**Table 1:** Geographic and climatic description (1980–2009) of the ten Swiss study sites (www.meteoswiss.admin.ch).

Station	Abbrev.	Latitude	Altitude	Aver. air temperature	Annual precipitation	Swiss domains	Climate change signals	
				+2 m			MAM	JJA
		(North)	(m.a.s.l.)	(°C)	(mm)		(Δ*T*_mean_)	(Δ*T*_mean_)
Basel	BAS	47°32′28″	316	10.4	838	CH-W	2.0	2.7
Bern	BER	46°59′27″	552	9.0	1066	CH-W	2.0	2.7
Buchs	BUC	47°23′04″	386	9.7	1040	CH-NE	2.0	2.6
Changins	CHA	46°24′04″	455	10.3	1007	CH-W	2.0	2.7
Chur	CHU	46°52′13″	556	9.6	855	CH-NE	2.0	2.6
Güttingen	GUE	47°36′06″	440	9.3	943	CH-NE	2.0	2.6
Magadino	MAG	46°09′36″	203	11.6	1813	CH-S	2.3	2.9
Sion	SIO	46°13′07″	482	10.0	609	CH-W	2.0	2.7
St. Gallen	STG	47°25′32″	775	8.3	1304	CH-NE	2.0	2.6
Wädenswil	WAE	47°13′14″	485	9.5	1387	CH-NE	2.0	2.6

Median climate change signals (2045–2074 vs. 1980–2009) of mean temperature (Δ*T*_mean_) were provided for the spring (MAM) as well for the summer (JJA) season (Hirschi *et al.* 2011). According to Fischer *et al.* (2011) three Swiss domains were defined for the aggregated climate change signals: northeastern (CH-NE), western (CH-W) and southern (CH-S) Switzerland.

Most of the ENSEMBLES simulations were performed in a transient mode from 1950 to 2100, and we selected 2045–2074 as the time frame for studying the effect of future climate conditions on pest phenology. We applied a stochastic weather generator (WG) [23] in combination with a re-sampling procedure [24] to downscale the seasonal and regional median climate change signal of the multi-model ensemble to the spatial and temporal scale of SOPRA [16]. The WG was used to examine the statistical structure of the observed weather and generate daily synthetic weather series of precipitation, solar radiation, temperature and temperature range consistent with this structure [25]. The selected Richardson-type WG is based on a Markov chain to model precipitation occurrence, gamma distribution for the precipitation amount and an autoregressive model for non-precipitation variables [26]. In a second step the daily weather series were downscaled into hourly weather data using a nearest neighbour re-sampling procedure [16]. For each day of produced by the daily WG, the ten most similar days were selected from the hourly station observations within a ±10-day time window around the corresponding day of year. Then one of the selected 10 days was randomly chosen. The similarity was quantified by daily mean precipitation and temperature, daily temperature range, and solar radiation. The WG and re-sampling procedure was calibrated for ten stations in Switzerland using observed hourly weather data from the reference period 1980–2009 (Table 1). For validation Codling Moth phenology derived by synthetic and observed weather was compared [16]. There was a very good agreement in day of year for early and late Codling Moth stages modelled from observed and synthetic weather data ([16]: Figure S1). Next, synthetic hourly weather series representative for the climate conditions of today and of the 2045–2074 time period (100 years in both cases) were generated and used to drive the Codling Moth model of the SOPRA system.

## Results

### Climate change scenario

The climate scenario adopted for the present study is summarised in Table 1 in terms of projected changes in mean annual Temperature (Δ*T*_mean_) for the spring and summer season and for the three areas. The reader is referred to [16] for a detailed discussion of the scenario, including mean precipitation and the seasonal aspects. In short, the median climate change signals (2045–2074 vs. 1980–2009) for the spring and summer seasons predict an increase in mean temperature between 2.0–2.7 (northern Switzerland) and 2.3–2.9 (southern Switzerland) (Table 1).

### Voltinism without changes in photoperiodic diapause induction

**Figure 1:**
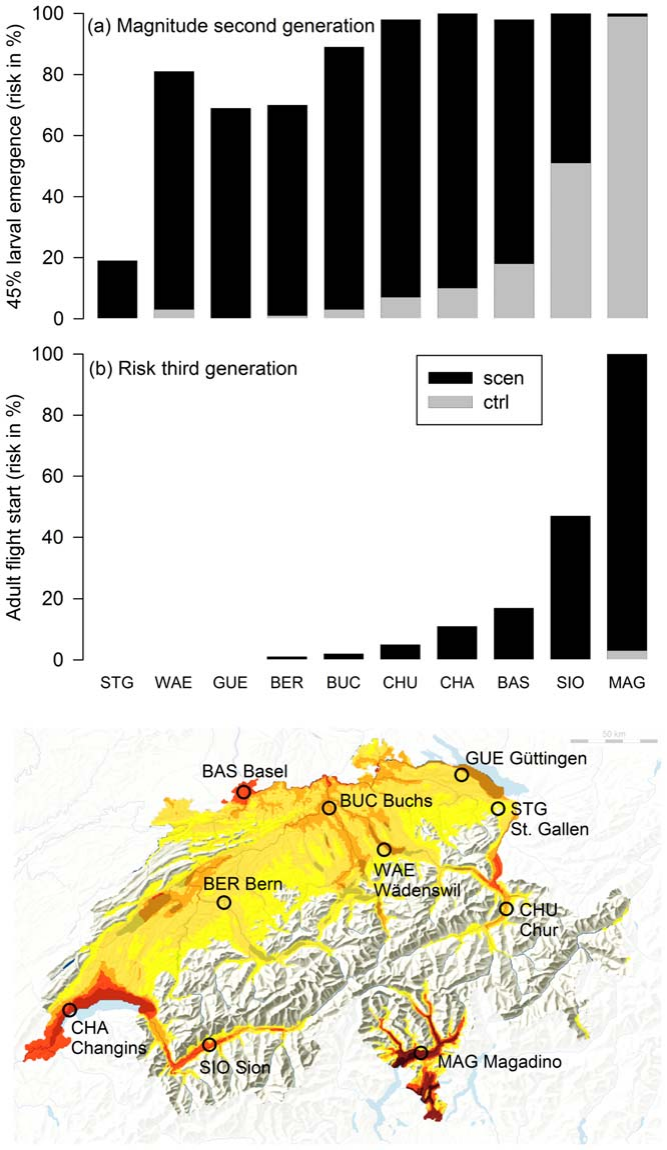
Codling Moth (*Cydia pomonella*) phenology under present and future climate conditions. Magnitude of the second Codling Moth generation (A) and the risk of a third generation (B) under present (ctrl) and future (scen) climate conditions at ten Swiss study sites (cf. Table 1). The magnitude of a second generation was measured as the probability of a 45% second generation larval emergence. The risk of a third generation was presented as the probability of a second generation adult flight start (>1%). Adult flight, oviposition and larval emergence are separated for first, second and third generations.

Under present climate and diapause induction conditions there is a 100% risk for overwintering adult flight at all sites. Furthermore there is a 100% risk for second generation (first generation adult flight) at the majority of the sites, with the exception of Wädenswil (97%), Bern (91%), Güttingen (88%), and St. Gallen (43%) (Table 1 for the sites). A 100% risk for a second generation larval emergence start is simulated for Basel, Changins, Buchs, Chur, Magadino and Sion while this risk is only 87% for Wädenswil, 78% for Bern and Güttingen, and 24% for St. Gallen. The risk of a pronounced second generation larval emergence (45% relative larval emergence) is below 20% at nearly all sites (Fig. 1a).

Under future climate conditions we simulate a 100% risk of a second generation and a pronounced second generation at all sites. The risk of a pronounced larval emergence (45% larval emergence) is between 70–100%, with the exception of St. Gallen (19%; Fig. 1a). The increase in the risk of a second generation is highly significant (Wilcoxon rank-sum test, *n* = 10, W = 19, *P* = 0.008). For present climate, we only identify a third generation at Magadino (second generation adult flight start; Fig. 1b). The risk of a third generation (second generation adult flight start) is highly variable considering future climate scenarios (Fig. 1b). The risk varies between 0% (Güttingen, St. Gallen, Wädenswil) and 100% (Magadino) (Fig. 1b). Additionally, third generation larval emergence may occur at some sites (Chur 2%, Basel and Changins 3%, Sion 22%, Magadino 97%). In general the increase in the risk of a third generation is highly significant (*n* = 10, W = 8, *P* = 0.002).

**Figure 2:**
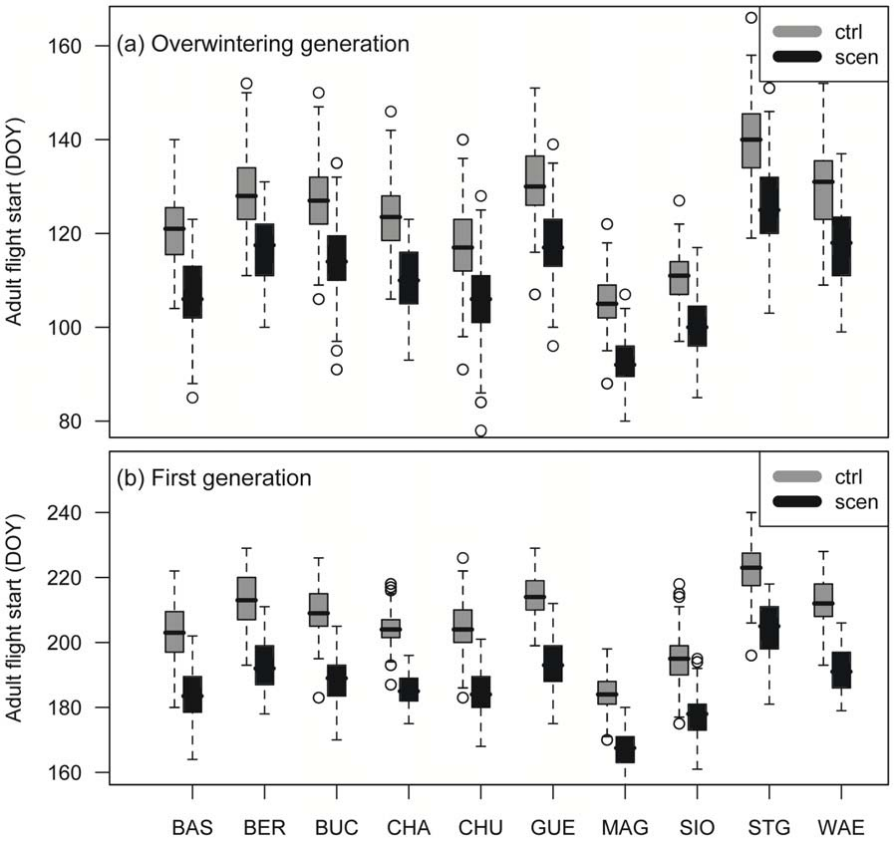
Codling Moth adult flight start under present and future climate conditions. Codling Moth day of year (DOY) of first generation (A) and second generation (B) adult flight start (>1%) under present (ctrl) and future (scen) climate conditions at ten Swiss study sites (see Table 1).

The increased probability for the development of an additional Codling Moth generation under changed climate is related to the shift in phenology to an earlier dates and to faster Codling Moth development during the season. In general, time of adult flight start of the overwintering generation changed from DOY 124±0.4 to 111±0.4 (mean±se; *n* = 100, *P*<0.0001 for all sites). Time of first generation adult flight start shifts from DOY 205±0.4 to 187±0.3 (*n* = 100, *P*<0.0001 for all sites). This approximately two-week adaptation in adult moth flight start is similar and significant for all study sites (*n* = 100, *P*<0.0001 for all sites; Fig. 2).

Under present climate, adult flight start of the overwintering generation is earliest at the Magadino site (DOY; mean±se; 105±1) and latest at the St. Gallen site (140±1). Additional 80 days are necessary for first generation adult moth flight start (Magadino: 185±1; St. Gallen: 222±1; Fig. 2). A second generation adult flight under present climate conditions was only identified at the Magadino site (222±1). Time of oviposition and larval emergence start for first generation ranged between 123±1/139±1 (Magadino) and 159±1/174±1 (St. Gallen). Second generation oviposition and larval emergence starts earliest at the Magadino site (193±1/200±1) and latest at the St. Gallen site (230±2/247±2; Table 2). The pest phenology model predicts the 45% larval emergence peak approximately 20 days after the larval emergence start. The identified two-week time-shift of adult moth flight start under changing climate was similar for other phenological stages such as oviposition, larval emergence start and 45% relative larval emergence of first and second generations, and significant in each case (*n* = 100, *P*<0.0001 for all stages and sites; Table 2). However there is an indication that the 45% relative larval emergence peak (and other stages of the second generation) may occur more than 14 days in advance under future climate conditions (Table 2).

**Table 2:** Codling Moth (*Cydia pomonella*) phenology under present (ctrl) and future climate (scen) at ten Swiss study sites (cf. Table 1) with day of year (DOY) of oviposition and larval emergence start (>1%) (*n* = 100; mean±se).

Station	Climate	First generation				Second generation			
		Oviposition	Larvae			Oviposition	Larvae		
		start	start	peak	(diff.)	start	start	peak	(diff.)
**BAS**	ctrl	137±1	153±1	181±1		213±1	222±1	239±2	
	scen	123±1	138±1	162±1	19	192±1	198±1	220±1	19
**BER**	ctrl	146±1	162±1	192±1		222±1	235±1	256±0	
	scen	133±1	148±1	173±1	19	202±1	209±1	230±1	16
**BUC**	ctrl	143±1	158±1	187±1		219±1	231±1	241±1	
	scen	129±1	144±1	169±1	18	197±1	204±1	226±1	15
**CHA**	ctrl	139±1	155±1	183±1		214±1	222±1	240±2	
	scen	126±1	141±1	165±1	18	193±1	199±1	221±1	19
**CHU**	ctrl	134±1	151±1	181±1		215±1	226±1	240±3	
	scen	123±1	137±1	163±1	18	193±1	200±1	224±1	16
**GUE**	ctrl	148±1	163±1	193±1		223±1	236±1	-	
	scen	133±1	148±1	174±1	19	202±1	209±1	231±1	-
**MAG**	ctrl	123±1	139±1	165±1		193±1	200±1	224±1	
	scen	109±1	124±1	148±1	17	174±1	180±1	200±1	24
**SIO**	ctrl	128±1	145±1	174±1		204±1	212±1	237±1	
	scen	116±1	133±1	159±1	15	185±1	192±1	214±1	23
**STG**	ctrl	158±1	174±1	205±1		230±2	247±3	-	
	scen	142±1	157±1	183±1	22	213±1	221±1	239±2	-
**WAE**	ctrl	147±1	162±1	190±1		221±1	232±1	244±5	
	scen	133±1	147±1	172±1	18	200±1	207±1	229±1	15

45% peak larval emergence and difference between ctrl and scen for peak larval emergence in DOY is given in parenthesis.

Codling Moth phenology was further analysed by considering the magnitude and length of the specific stages. The magnitude (peak relative phenology) of the specific stages is similar under present and future climate conditions for early stages up to the first generation larvae (*n* = 100, *P*>0.05; Table 3). There is a significant increase in the magnitude of the first generation pupae and all later stages under future climate conditions (*n* = 100, *P*<0.0001; Table 3). The magnitude of the second generation larvae nearly doubled under future climate (exception the currently already warm sites Magadino and Sion; Table 3). The length of earlier stages such as overwintering adults, first generation oviposition and larval emergence is shortened (in DOY; Table 4). In contrast, first generation pupae and adult flight, and later stages such as second generation oviposition and larval emergence is prolonged in the future at all sites except Magadino and Sion (Table 4). At the Magadino and Sion site there is a risk (22%, 97%, respectively) for the development of third generation larvae (with a magnitude of 0.16±0.01 at Magadino and 0.10±0.03 at Sion). The development of a third generation explains the finding that at these two sites there is no prolonged second generation and an increase in the magnitude of later stages. The overlap of different stages is directly related to their length. We assessed the overlap of first and second generation larval emergence, as well as the overlap of overwintering and first generation adult flight. We identified a significant decrease in the length of overlap of first and second generation larval emergence (*n* = 100, *P*<0.0001). The shortened first generation larval emergence under future climate mainly explains this finding. The prolonged second generation larval emergence is at the end and does not affect the overlap with first generation larval emergence. We have not identified a significant change in overlap of adult flight (*n* = 100, *P*>0.0001).

**Table 3:** Codling Moth (*Cydia pomonella*) phenology under present (ctrl) and future climate (scen) at ten Swiss study sites (cf. Table 1) as relative proportion of the population at the peak relative phenology of the specific stages (*n* = 100; mean±se).

Station	Climate	First generation				Second generation	
		Oviposition	Larvae	Pupae	Adults	Oviposition	Larvae
**BAS**	ctrl	0.23±0.00	0.79±0.00	0.27±0.01	0.25±0.01	0.12±0.01	0.35±0.02
	scen	0.23±0.00	0.79±0.00	0.37±0.00	0.47±0.00	0.20±0.00	0.67±0.01
**BER**	ctrl	0.22±0.00	0.77±0.00	0.14±0.01	0.14±0.01	0.07±0.01	0.21±0.02
	scen	0.22±0.00	0.77±0.00	0.34±0.00	0.39±0.01	0.17±0.00	0.52±0.01
**BUC**	ctrl	0.23±0.00	0.79±0.00	0.19±0.01	0.17±0.01	0.09±0.00	0.29±0.02
	scen	0.23±0.00	0.80±0.00	0.37±0.00	0.45±0.00	0.20±0.00	0.61±0.01
**CHA**	ctrl	0.21±0.00	0.76±0.00	0.24±0.01	0.24±0.01	0.12±0.00	0.31±0.01
	scen	0.22±0.00	0.76±0.00	0.34±0.00	0.44±0.00	0.19±0.00	0.65±0.00
**CHU**	ctrl	0.21±0.00	0.76±0.00	0.22±0.01	0.22±0.01	0.11±0.00	0.33±0.02
	scen	0.22±0.00	0.76±0.00	0.34±0.00	0.44±0.00	0.19±0.00	0.63±0.01
**GUE**	ctrl	0.23±0.00	0.78±0.00	0.14±0.01	0.11±0.01	0.06±0.00	0.20±0.03
	scen	0.23±0.00	0.78±0.00	0.35±0.00	0.39±0.01	0.17±0.00	0.51±0.01
**MAG**	ctrl	0.22±0.00	0.75±0.00	0.33±0.00	0.43±0.00	0.18±0.00	0.63±0.01
	scen	0.23±0.00	0.77±0.00	0.35±0.00	0.45±0.00	0.18±0.00	0.69±0.00
**SIO**	ctrl	0.22±0.00	0.73±0.00	0.30±0.00	0.34±0.01	0.15±0.00	0.44±0.01
	scen	0.22±0.00	0.74±0.00	0.32±0.00	0.42±0.00	0.18±0.00	0.66±0.00
**STG**	ctrl	0.24±0.00	0.80±0.00	0.07±0.01	0.08±0.02	0.05±0.01	0.20±0.00
	scen	0.23±0.00	0.79±0.00	0.27±0.02	0.24±0.01	0.12±0.01	0.32±0.02
**WAE**	ctrl	0.23±0.00	0.78±0.00	0.17±0.01	0.14±0.01	0.07±0.01	0.22±0.03
	scen	0.23±0.00	0.78±0.00	0.35±0.00	0.42±0.01	0.18±0.00	0.56±0.01

**Table 4:** Codling Moth (*Cydia pomonella*) phenology under present (ctrl) and future climate (scen) at ten Swiss study sites (cf. Table 1) as length (in days) of the specific stages (*n* = 100; mean±se).

Station	Climate	First generation					Second generation		
		Oviposition	Larvae	(diff.)	Pupae	Adults	Oviposition	Larvae	(diff.)
**BAS**	ctrl	38.5±0.8	51.9±0.6		25.4±1.0	24.7±0.8	22.4±0.7	38.8±1.0	
	scen	32.8±0.6	46.1±0.6	5.8	29.3±0.3	31.5±0.3	26.4±0.4	38.3±0.4	0.5
**BER**	ctrl	42.1±0.8	55.6±0.7		17.5±0.9	20.7±0.8	18.0±0.8	39.6±2.8	
	scen	34.2±0.7	46.8±0.5	8.8	29.3±0.5	30.0±0.6	26.0±0.5	37.7±0.6	1.9
**BUC**	ctrl	40.4±0.7	53.0±0.6		20.7±0.8	21.0±0.8	19.6±0.7	43.0±2.0	
	scen	33.8±0.6	46.3±0.6	6.7	30.0±0.4	32.1±0.4	27.4±0.3	38.9±0.5	4.1
**CHA**	ctrl	36.2±0.7	51.0±0.6		23.7±0.8	23.9±0.6	21.5±0.5	37.0±1.0	
	scen	30.6±0.5	44.9±0.6	6.1	28.2±0.4	31.5±0.3	26.0±0.3	37.8±0.4	−0.8
**CHU**	ctrl	37.6±0.8	56.2±0.7		23.8±0.9	25.2±0.7	22.8±0.7	44.4±1.4	
	scen	30.4±0.6	48.3±0.7	7.9	30.7±0.5	34.0±0.4	28.4±0.4	40.6±0.5	3.8
**GUE**	ctrl	41.9±0.8	54.9±0.7		17.3±1.0	18.1±0.8	16.6±0.8	43.0±3.6	
	scen	33.8±0.7	47.0±0.6	7.9	30.3±0.5	30.1±0.5	25.7±0.5	37.8±0.6	5.2
**MAG**	ctrl	31.4±0.6	47.9±0.5		29.6±0.4	33.1±0.3	27.0±0.2	40.2±0.4	
	scen	30.0±0.6	44.4±0.5	3.5	26.3±0.3	28.9±0.3	23.4±0.3	34.5±0.2	5.7
**SIO**	ctrl	32.6±0.7	52.0±0.6		29.1±0.6	30.5±0.6	26.3±0.5	41.5±0.6	
	scen	29.5±0.6	46.8±0.6	5.2	26.4±0.4	31.5±0.3	25.2±0.4	39.1±0.4	2.4
**STG**	ctrl	45.2±0.8	59.2±0.9		12.7±1.4	17.1±1.9	16.4±2.6	35.0±0.0	
	scen	38.6±0.8	48.2±0.6	11	25.5±1.1	23.6±0.7	20.9±0.7	36.4±1.1	−1.4
**WAE**	ctrl	40.6±0.8	53.3±0.7		19.3±1.0	19.5±0.8	17.7±0.8	35.6±2.0	
	scen	34.4±0.6	45.6±0.6	7.7	29.8±0.6	30.7±0.5	26.6±0.4	37.0±0.5	−1.4

For larval emergence length the difference between ctrl. and scen in days is given in parenthesis.

### Sensitivity of future phenology to photoperiodic diapause induction

**Figure 3:**
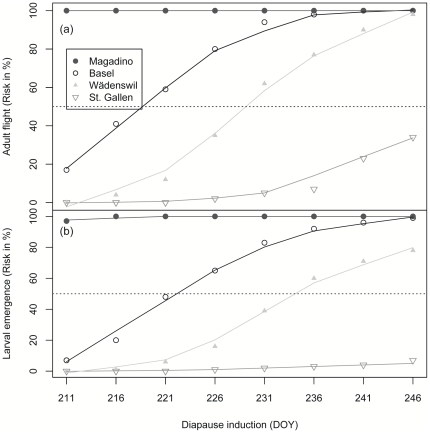
Sensitivity of Codling Moth phenology to photoperiodic diapause induction. Risk of second generation adult flight start and third generation larval emergence start. Diapause induction was changed from the present day of year (DOY 211, End of July) to DOY 246 (Beginning of September).

Global warming may further induce a shift in voltinism based on changes in life-history traits such as diapause induction. At Magadino there is already 100% risk of a second generation adult flight start under the present diapause induction date (Fig. 3a). A shift in diapause induction to later dates will therefore not increase the future risk of an additional third generation. In contrast, the future risk proves to be rather sensitive to the diapause induction date at Basel, where a 50% and 100% risk of a second generation adult flight start is simulated by a 10 and 20 day delay in diapause induction. At Wädenswil a shift of 20 and 35 days is necessary for a 50% and a 100% risk of a second generation adult flight start. The future risk of second generation adult flight is below 50% for all diapause induction dates simulated at St. Gallen. In general a shift in diapause induction between 25 and 35 days is necessary to simulate a 100% risk of a third generation at most of the study sites (Fig. 3a).

A 50% and 100% risk for a third generation larval emergence start at the Basel site is simulated by 10 and 35 days delay in diapause induction (Fig. 3b). At the Wädenswil site a 50% risk of third generation larval emergence is induced by a 25-day shift in diapause induction. There is no simulated 100% risk for third generation larval emergence start at the Wädenswil site even when postponing diapause induction by 5 weeks (DOY 246). At the St. Gallen site the risk remains below 50% for the whole range of simulated diapause induction dates. A simulated five-week shift in diapause induction revealed no general 100% risk of third generation larval emergence start. A shift in diapause induction between 10–25 days is necessary for a 50% risk of third generation larval emergence. Considering the DOY of the 100% risk for the second generation adult flight start, an additional shift in diapause induction of 5–10 days is necessary for the 100% risk of the third generation larval emergence start (Fig. 3a–b). There is an indication of a linear relationship between time of diapause induction (DOY) and the risk of the development of a third generation at earlier DOY for some but not for all sites (Fig. 3a–b).

## Discussion

An increased number of generations in multivoltine arthropod species are expected as a result of shorter development times under changed climatic conditions [5]. Consistent with this global trend, the present study identified significant changes in Codling Moth phenology as a consequence of changing climate in Swiss apple orchards. Presently, under conditions characteristic of northern Europe, strong second generations only occur in the warmest regions and exceptional warm years. Under future climatic conditions, a pronounced second generation is expected in most habitats of the species, with a non-negligible risk for an additional third generation larvae as appearing at almost all sites investigated across Switzerland (up to 97%). Other studies have shown that a similar effect of global warming is expected for voltinism in Codling Moth populations from walnut trees [4]. Additionally we could show that Codling Moth will profit by local adaptation of photoperiodic diapause induction thereby further extending its development season according to the elevated temperatures late in season.

Voltinism is under genetic and environmental control [27] and thereby under constant selection pressure. Conditions that would favour univoltinism are the proportion of obligate univoltine individuals, the disruption of the developmental synchrony associated with multivoltinism and host plant phenology, intensive larval competition and limited food supply [13]. Experimental results in northern Swiss orchards (approx. 500 m a.sl.) revealed that approximately 40–50% of the individuals have an obligate univoltine life-cycle [28]. These results are similar for studies in northern latitudes, with percentage of univoltine individuals increasing from 12% at southern latitudes to 76% at northern latitudes [29], [30]. The proportion of obligate univoltine individuals is lower in southern (10–20%) compared to northern Switzerland (50%) (Stoeckli *et al.*, unpuplished data). The impact of climate change on Codling Moth phenology may be amplified by an evolutionary change from uni- to multivoltine populations, which is especially relevant for the northern Switzerland with temperate climate and a higher proportion of obligate univoltine individuals.

A shift in critical photoperiods for diapause induction with ongoing climate change was already observed in nature. A decline in the critical photoperiod from 15.79 to 15.19 h light (1972–1996) was identified in the pitcher plant mosquito [10], whereas critical photoperiod was shortened by 30 min (1991–2002) in a water strider [31]. The critical photoperiod shortened by 14 min (1995–2002) in the lepidopteran fall webworm [32]. Bradshaw & Holzapfel (2001) concluded that the decline in the critical photoperiod of the pitcher plant mosquito corresponds to 2–3 days per decade, which is similar to the shift occurring with other phenological stages, such as earlier oviposition in birds or the advancement of flowering phenology [10]. The corresponding genetic change in a life-history trait may induce range shifts in species. A one hour decline in critical photoperiod corresponds to a southern movement of five degrees latitude for many insect species [33]. For Codling Moth, a decrease of the critical photoperiod by 7.2 min was simulated for each 1°C increase in temperature [34]. Accordingly, field observations indicate that diapause induction in Codling Moth changes along with climatic clines and shifts in host- and geographic range. In Eastern Europe critical photoperiod and voltinism changes strongly along with average temperatures [30]. A linear cline in the critical photoperiod of Codling Moth was formed along with geographic range shift after its establishment in North America [13], [35]. A phylogenetic decrease in the photophase from 15 h to 13 h along with the formation of host-races on apple, walnut and plum in California additionally supports our simulated results [36]. Intraspecific plasticity may further promote genetic changes in life-history traits. The yearly variation in the critical photoperiod up to two weeks in a local Codling Moth population underlines possible adaptations in photoperiodic diapause induction [13].

Host-plant phenology can be important to assess changes in pest severity as a response of climate change. For example, there is an ongoing change in flowering phenology to an earlier start of bloom [37], [38]. But the effect of this is (and will be) largely restricted on insects that are synchronized with flowering early in season (e.g. the winter moth [39]). For Codling Moth the identified two-week shift to earlier dates in phenological events, such as adult flight start, is in line with observed and predicted earlier leaf unfolding and later leaf senescence (prolongation of the growing season) and the availability of leaves and fruits for egg deposition and larval development [3], [40], [41]. Later on in season, there may indeed be a discrepancy between herbivores and fruit development due to advanced time of harvest. But as for selection against later Codling Moth generations, on the long term, climate change will also lead to planting of fruit varieties that are adapted to a longer season – as it is the case in the Mediterranean Apple growing regions.

It is evident, that the possible shift in Codling Moth phenology will require adaptations of plant protection strategies to maintain their sustainability. The most suggested strategy for commercial orchards in northern pre-alpine regions is based on pheromone mating disruption using codlemone dispensers [15]. With increasing temperature the present amounts of pheromone in some types of dispensers will most likely be insufficient to cover the entire season, and the dispenser load may have to be increased or new carriers have to be used. Mating disruption alone may also no longer be sufficient to control Codling Moth in important production areas, because during multiple generations higher population densities may build up. The sole application of granulosis viruses, suggested for organic farms that are not suitable for mating disruption, will most likely no longer be the option of choice, due to the increased risk of resistances [42] so that other components have to be included in organic control strategies (e.g., organic insecticides, summer oil treatments etc.). In integrated production schemes, alternatively insect growth regulators (IGRs) may have to be combined with pheromone mating disruption in future, which, however, would not be an option for organic agriculture. Also, the repeated treatment with the same group of active ingredients of IGRs may provoke the development of resistances, as it has been previously the case [12], [43], [44]. Therefore, every integrated strategy will probably have to be supported by new products with new modes of actions, but in any case will need specific monitoring and precisely timed application as assured by decision support systems like SOPRA [17]. As a consequence of ongoing climatic change, more research will be necessary to allow for sustainable apple production in most temperate regions of the world in future.

## Supporting Information

Figure S1 **Validation of seasonal phenology from SOPRA with field observations.** Observed weather was used to compare seasonal relative phenology (%) for adult flight (overwintering and first generation) from SOPRA output and trap catches of adult male moths (traps were checked once per week) at the climate station Wädenswil in 2003. The year 2003 was taken as reference to present the good accordance between SOPRA output and field observations for early and additionally also later adult flight.
(TIF)

Figure S2 **Validation of DOY for first flight from SOPRA with field observations.** SOPRA was driven with observed weather to compare the DOY for adult moth first flight (overwintering generation) with field observations (trap catches of adult male moths) at the climate station Wädenswil between 1990 and 2010. There was a significant correlation (Pearson's product correlation; *P* = 0.03, *r* = 0.5), indicating that the SOPRA model is accurate under variable climate conditions. On average, first male moths were caught on May 14, which was 5 days later compared to the output from SOPRA ([16]: Fig. 3).
(TIF)
