# Supplementary figures and images for: Impact of Climate Change on Voltinism and Prospective Diapause Induction of a Global Pest Insect – Cydia pomonella (L.)
Source: PLoS One. 2012 Apr 23;7(4):e35723. doi: 10.1371/journal.pone.0035723 (PMC3335082; doi:10.1371/journal.pone.0035723)

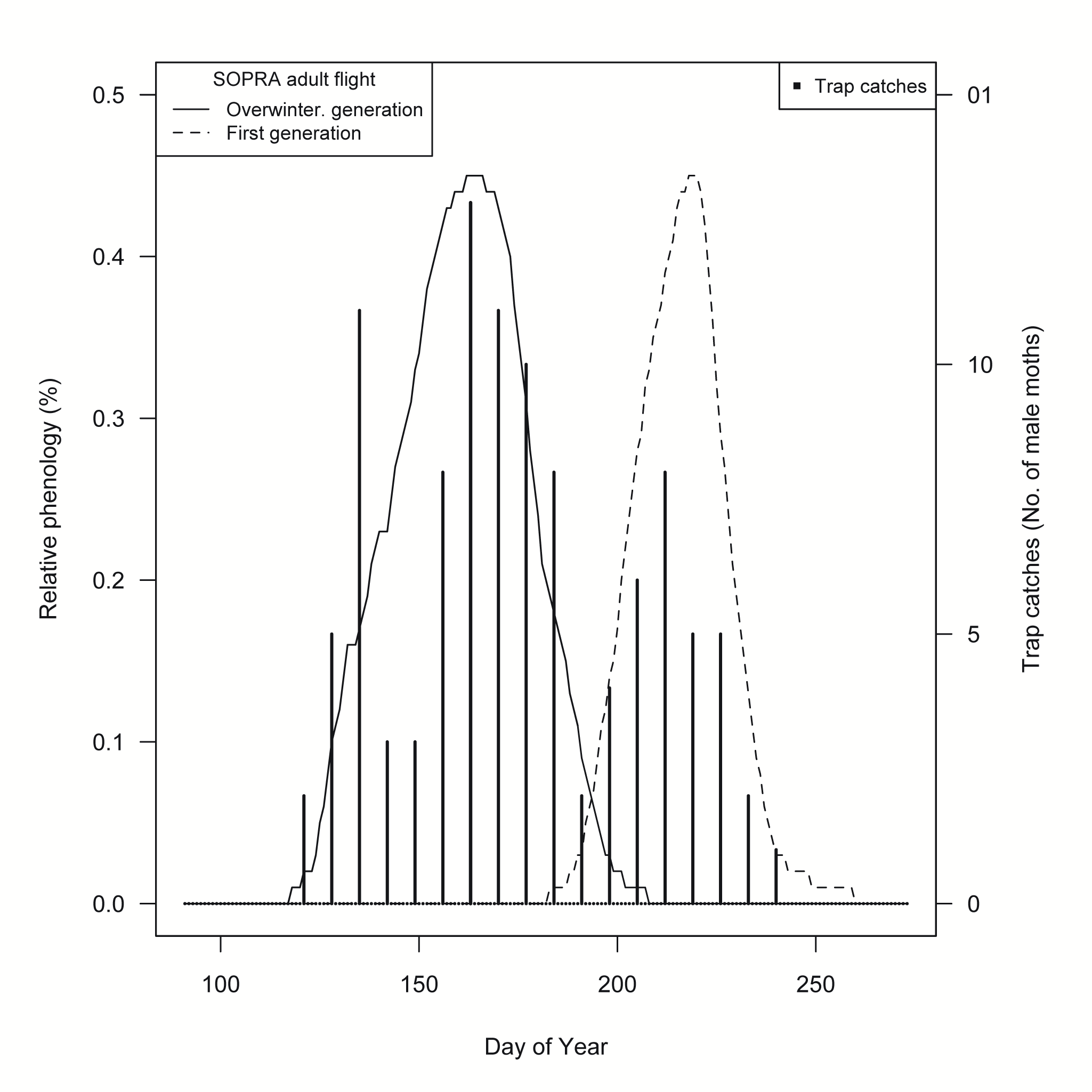

Supplement: Figure S1 — Validation of seasonal phenology from SOPRA with field observations. Observed weather was used to compare seasonal relative phenology (%) for adult flight (overwintering and first generation) from SOPRA output and trap catches of adult male moths (traps were checked once per week) at the climate station Wädenswil in 2003. The year 2003 was taken as reference to present the good accordance between SOPRA output and field observations for early and additionally also later adult flight. (TIF) [file pone.0035723.s001.tif]

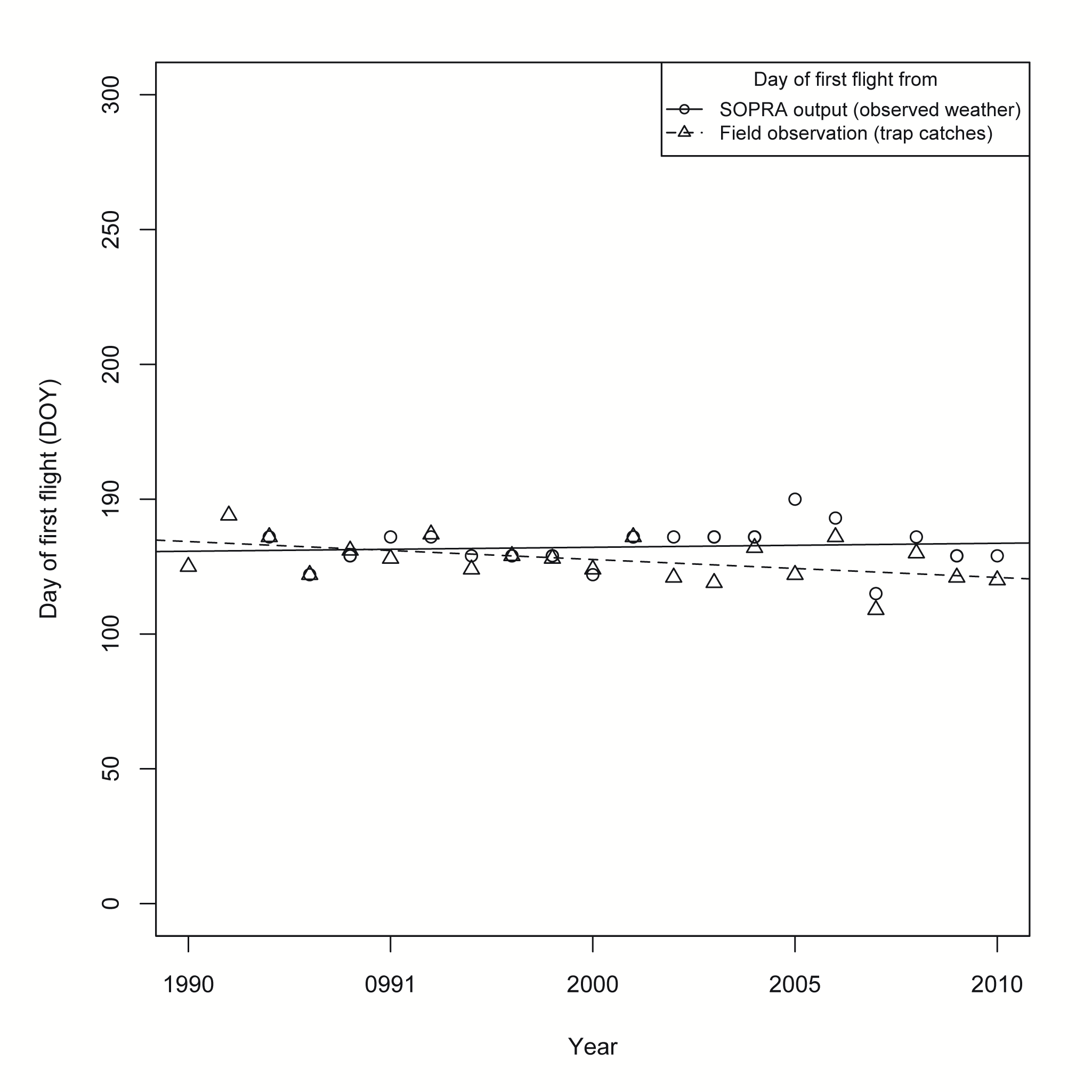

Supplement: Figure S2 — Validation of DOY for first flight from SOPRA with field observations. SOPRA was driven with observed weather to compare the DOY for adult moth first flight (overwintering generation) with field observations (trap catches of adult male moths) at the climate station Wädenswil between 1990 and 2010. There was a significant correlation (Pearson's product correlation; P = 0.03, r = 0.5), indicating that the SOPRA model is accurate under variable climate conditions. On average, first male moths were caught on May 14, which was 5 days later compared to the output from SOPRA ([16]: Fig. 3). (TIF) [file pone.0035723.s002.tif]
